# Zinc oxide nanoparticles prepared through microbial mediated synthesis for therapeutic applications: a possible alternative for plants

**DOI:** 10.3389/fmicb.2023.1227951

**Published:** 2023-09-05

**Authors:** Mahadevamurthy Murali, H. G. Gowtham, N. Shilpa, S. Brijesh Singh, Mohammed Aiyaz, R. Z. Sayyed, Chandan Shivamallu, Raghu Ram Achar, Ekaterina Silina, Victor Stupin, Natalia Manturova, Ali A. Shati, Mohammad Y. Alfaifi, Serag Eldin I. Elbehairi, Shiva Prasad Kollur

**Affiliations:** ^1^Department of Studies in Botany, University of Mysore, Mysuru, India; ^2^Department of PG Studies in Biotechnology, Nrupathunga University, Bangalore, India; ^3^Department of Studies in Microbiology, University of Mysore, Mysuru, India; ^4^Department of Studies in Biotechnology, University of Mysore, Mysuru, India; ^5^Department of Microbiology, PSGVP Mandal’s S I Patil Arts, G B Patel Science and STKV Sangh Commerce College, Shahada, India; ^6^Department of Biotechnology and Bioinformatics, School of Life Sciences, JSS Academy of Higher Education & Research, Myuru, India; ^7^Division of Biochemistry, School of Life Sciences, JSS Academy of Higher Education and Research, Mysuru, India; ^8^Department of Human Pathology, I.M. Sechenov First Moscow State Medical University (Sechenov University), Moscow, Russia; ^9^Department of Surgery, Pirogov Russian National Research Medical University (RNRMU), Moscow, Russia; ^10^Biology Department, Faculty of Science, King Khalid University, Abha, Saudi Arabia; ^11^School of Physical Sciences, Amrita Vishwa Vidyapeetham, Mysuru, India

**Keywords:** biological applications, green synthesis, microbial synthesis, nanoparticles, toxicological effects, zinc oxide

## Abstract

Zinc oxide nanoparticles (ZnO-NPs) synthesized through biogenic methods have gained significant attention due to their unique properties and potential applications in various biological fields. Unlike chemical and physical approaches that may lead to environmental pollution, biogenic synthesis offers a greener alternative, minimizing hazardous environmental impacts. During biogenic synthesis, metabolites present in the biotic sources (like plants and microbes) serve as bio-reductants and bio-stabilizers. Among the biotic sources, microbes have emerged as a promising option for ZnO-NPs synthesis due to their numerous advantages, such as being environmentally friendly, non-toxic, biodegradable, and biocompatible. Various microbes like bacteria, actinomycetes, fungi, and yeast can be employed to synthesize ZnO-NPs. The synthesis can occur either intracellularly, within the microbial cells, or extracellularly, using proteins, enzymes, and other biomolecules secreted by the microbes. The main key advantage of biogenic synthesis is manipulating the reaction conditions to optimize the preferred shape and size of the ZnO-NPs. This control over the synthesis process allows tailoring the NPs for specific applications in various fields, including medicine, agriculture, environmental remediation, and more. Some potential applications include drug delivery systems, antibacterial agents, bioimaging, biosensors, and nano-fertilizers for improved crop growth. While the green synthesis of ZnO-NPs through microbes offers numerous benefits, it is essential to assess their toxicological effects, a critical aspect that requires thorough investigation to ensure their safe use in various applications. Overall, the presented review highlights the mechanism of biogenic synthesis of ZnO-NPs using microbes and their exploration of potential applications while emphasizing the importance of studying their toxicological effects to ensure a viable and environmentally friendly green strategy.

## 1. Introduction

Recently, the ZnO-NPs have gained recognition for their application in several industrial areas, including pharmaceuticals, food, photocatalyst, cosmetics, and agriculture, due to their distinctive properties (Jain et al., 2020; Murali et al., 2021a; Akhtar et al., 2022). As mentioned earlier, the large surface area-to-volume ratio of ZnO-NPs makes them more efficient than their counterparts, i.e., bulk ZnO, for their applications. Physical and chemical techniques have traditionally been employed for synthesizing ZnO-NPs, which provide high production rates with control sizes. However, the physical approach uses much energy, pressure, and heat, whereas the chemical method uses toxic and hazardous chemicals that contribute to environmental pollution and adversely affect human and animal health (Basnet et al., 2018). Moreover, the chemically synthesized ZnO-NPs have limited clinical and biological applications because they are toxic, less biocompatible, and may also bind or reside in the final NP products that interfere with the biological applications (Anjum et al., 2021). Hence, it is necessary to develop non-toxic, bio-safe, cost-effective, more eco-friendly, biocompatible ZnO-NPs as a substitute.

The green synthesis of ZnO-NPs, which uses diverse bioactive chemicals from plants and microbes as reductants and stabilizers, has recently come into the spotlight as a replacement for chemically and physically manufactured ZnO-NPs without altering their properties. Apart from plants, both unicellular and multicellular organisms (such as bacteria, actinomycetes, fungi, and yeast) are involved in the biological synthesis of ZnO-NPs (Kundu et al., 2014; Moghaddam et al., 2017; Shamsuzzaman et al., 2017; Kalaba et al., 2021). The green synthesis process is devoid of toxic chemicals and materials, energy-efficient, eco-friendly, and makes their applications in living organisms completely safe (Weldegebrieal, 2020). The plant-mediated synthesis of ZnO-NPs has been extensively reviewed to understand their unique properties and biological applications (Ahmed et al., 2017; Murali et al., 2021a); in contrast, only a few studies have noted the importance of microbial synthesis of ZnO-NPs and their applications. Hence the present study was focused on providing a holistic view of the microbe-mediated synthesis of ZnO-NPs and their potential applications, with specific objectives that include synthesis mechanisms and biological properties apart from their toxicological effects on the environmental ecosystem.

## 2. Microbes for ZnO-NP synthesis

Since the microbes are easily reproducible compared to plants, it offers an advantage over plant-mediated ZnO-NP synthesis. In the new millennium, using microbes, *viz.,* bacteria, actinomycetes, fungi and yeast, has attracted considerable interest in synthesizing the ZnO-NPs. These organisms serve as miniature nano-factories wherein the enzymes, proteins or biomolecules secreted by them help to selectively reduce metal ions into their corresponding metal or metal oxide NPs. The formation of mono- and poly-dispersed NPs with different sizes and shapes is attributed to the numerous organic compounds released into the growing media or suspension cultures (Yusof et al., 2020a). Not all microbes are involved in the NPs synthesis process due to unique enzyme activity and metabolic processes, which differ from organism to organism. Due to these properties, selecting appropriate microbes independent of their enzyme activity or metabolic and biosynthetic pathways (intracellular or extracellular) is critical for forming ZnO-NPs, which remains unexplored. In addition, these microbes must also be able to tolerate heavy metals to synthesize the ZnO-NPs, as it is noted that the high metal stress is well documented to alter various microbial activities (Jain et al., 2020). Under this stress, the microbes reduce metal ions into appropriate metal NPs, demonstrating their potential to act as natural nano-factories (Lahiri et al., 2021). Microbes generally inhabit metal-rich environments and have strong metal resistance owing to their metal chelation and adsorption by intracellular and extracellular proteins (Yusof et al., 2019). As a result, mimicking the natural biomineralization process for synthesizing ZnO-NPs could be considered a promising strategy. Many Zn metal-tolerating microbes have been isolated from native metal-rich soils and mines to synthesize the ZnO-NPs (Jain et al., 2013).

In addition, metal (Zn) precursors (such as zinc acetate, zinc chloride, zinc nitrate, zinc sulphate, etc.) are needed for the microbe-mediated ZnO-NP synthesis. These salts are typically provided as soluble salts precipitating in the microbial cell suspension or its extract with bioactive components during the synthesis, usually taking minutes or hours (Murali et al., 2021b). The successful transformation is indicated by precipitation/ white deposition or gradual changes in the suspension color. Once the synthesis process is complete, the mixture contains microbial cells and ZnO-NPs, separated using various techniques, including centrifugation, filtration, or calcination. In addition, to obtain white crystalline powder of ZnO-NPs, the obtained particles are dried in a hot air oven for an extended time at 60°C after being completely washed with distilled water, followed by ethanol to remove any contaminants that were present (Kundu et al., 2014; Kalpana et al., 2018; Yusof et al., 2019). Different physicochemical methods characterize the synthesized NPs to ascertain their distinctive characteristics, such as shape, particle size, purity, composition, surface charge, and active functional groups. These methods include Ultraviolet–Visible (UV–Vis) spectroscopy, Fourier Transform Infrared (FTIR) spectroscopy, X-Ray Diffraction (XRD), Dynamic Light Scattering (DLS), Scanning Electron and Transmission Electron Microscopy (Faridvand et al., 2021; Nayeri et al., 2021; Thakur et al., 2022). Furthermore, several factors, like temperature, pH, reaction period and precursor concentration, are essential for determining the production rate, yield, and morphologies of NPs. The microbe-assisted synthesis of ZnO-NPs and their potential biological applications are listed in Table 1.

**Table 1:** Microbe-mediated ZnO-NPs and their potential biological applications.

Microbes	Precursor	Size (nm)	Shape	Purification methods	Application	Reference
Bacteria						
*Aeromonas hydrophila*	Zinc oxide	57	Spherical/oval	Centrifugation	Antimicrobial	Jayaseelan et al. (2012)
*Lactobacillus sporogens*	Zinc sulphate	145.7	Hexagonal	Centrifugation, washing and drying in oven at 40°C for 8 h	Antibacterial	Mishra et al. (2013)
*Serratia ureilytica*	Zinc acetate	170–600	Spherical	–		Dhandapani et al. (2014)
*Rhodococcus pyridinivorans*	ZnSO_4_.H_2_O	100–120	Roughly spherical	Centrifugation, filtration, washing and drying at 70°C in hot air oven for 4 h	Antibacterial, Anticancer, Photocatalytic and Drug delivery	Kundu et al. (2014)
*Pseudomonas aeruginosa*	Zinc nitrite	35–80	Spherical	Centrifugation, washing and drying at 70°C under vacuum oven	Antioxidant	Singh et al. (2014)
*Bacillus licheniformis*	Zinc acetate dihydrate	320–400	Rod-like	Centrifugation	Photocatalytic	Tripathi et al. (2014)
*Acinetobacter schindleri*	Zinc nitrate	20–100	Polydispersed and spherical	Centrifugation, washing and drying in hot air oven at 60°C for 6 h	Antibacterial	Busi et al. (2016)
*Streptomyces* sp.	Zinc chloride	20–50	Spherical	–	Antibacterial and anticancer	Balraj et al. (2017)
*Sphingobacterium thalpophilum*	Zinc nitrate	112	Triangle chips-like	Drying at 120°C and annealing at 700°C for 5 h	Antibacterial	Rajabairavi et al. (2017)
*Staphylococcus aureus*	Zinc acetate	10–50	Acicular	Centrifugation, washing, lyophilization and storage at 0°C		Rauf et al. (2017)
*P. aeruginosa*	Zinc nitrate	50–100	Pseudospherical	–	Antimicrobial	Barsainya and Singh (2018)
*L. paracasei*	Zinc nitrate	1,179	Spherical	–	Antibacterial	Król et al. (2018)
*B. megaterium*	Zinc nitrate	45–95	Rod and cubic	Centrifugation and repeated washing		Saravanan et al. (2018)
*S. enissocaesilis*	Zinc sulfate	5–20	Spherical	Centrifugation, washing and drying		Shaaban and El-Mahdy (2018)
*Halomonas elongata*	Zinc chloride	18	Spherical	–		Taran et al. (2018)
*P. putida*	Zinc nitrate	44.5	Spherical	Centrifugation and drying in hot air furnace 400°C for 2 h		Jayabalan et al. (2019)
*B. haynesii*	Zinc sulphate	50	Spherical	Drying at 80°C and annealing at 700°C for 5 h		Rehman et al. (2019)
*B. cereus*	Zinc sulphate hepta-hydrate	58–63	Irregular	–	Antibacterial	Iqtedar et al. (2020)
*S. nematodiphila*	Zinc sulfate	15–30	Roughly spherical	Washing through multiple centrifugations and drying at 120°C	Antimicrobial and Photocatalytic	Jain et al. (2020)
*L. plantarum*	Zinc nitrate	191–291	Flower-like and irregular	Cell biomass: Ultrasonic disruption at 30°C for 30 min, centrifugation and drying at 100°C. Cell-free supernatant: Centrifugation, washing and drying at 100°C	Antibacterial	Yusof et al. (2020b)
*B. cereus*	Zinc sulfate heptahydrate	21–35	Spherical	Centrifugation, washing and freeze-drying		Ahmed et al. (2021)
*Lactobacillus* spp.	Zinc acetate	32	Spherical	Interruption by ultrasound cycles in 100 W for 5 min, centrifugation, washing and air-drying at 60°C	Antimicrobial and anticancer	Suba et al. (2021)
Actinomycetes						
*Streptomyces* sp.	Zinc acetate dihydrate	16–25	Spherical	Centrifugation, drying at 120°C overnight and heating at 450°C for 5 h	Antibacterial, and anticancer	Shanmugasundaram and Balagurunathan (2017)
*S. plicatus*	Zinc sulfate	21.72	Spherical	Centrifugation, washing and drying in an oven at 90°C	Antimicrobial	Kalaba et al. (2021)
*Pseudochrobactrum* sp.	Zinc acetate	90–110	Granular	Oven drying at 85°C, calcination for 7 h in muffle furnace at 700°C and grinding	Photocatalytic	Siddique et al. (2021)
*S. enisocaesilis*	Zinc sulphate	12–326	Needle shape	Washing, centrifugation and drying in muffle furnace at 400°C for 8 h	Antibacterial	Rajivgandhi et al. (2022)
Fungi						
*Aspergillus terreus*	Zinc sulfate	54.8–82.6	Spherical	Centrifugation and lyophilization	Antifungal	Baskar et al. (2013)
*A. aeneus*	Zinc acetate	100–140	Spherical	–	–	Jain et al. (2013)
*A. fumigatus*	Zinc sulfate	60–80	Spherical	Centrifugation and lyophilization	Antibacterial	Rajan et al. (2016)
*A. niger*	Zinc chloride	41–75	Spherical	Centrifugation and drying in oven at 60°C		Ibrahem et al. (2017)
*Candida albicans*	Zinc oxide	∼20	Quasi-spherical	Centrifugation	–	Shamsuzzaman et al. (2017)
*A. terreus*	Zinc acetate	10–45	Spherical	Separation and drying at 150°C for 48 h	Antibacterial and anticancer	Fouda et al. (2018)
*A. niger*	Zinc nitrate	53–69	Spherical	Centrifugation	Antibacterial and photocatalytic	Kalpana et al. (2018)
*A. niger*	Zinc nitrate	30–70	Spherical	–	Antioxidant and anticancer	Es-haghi et al. (2019)
*A. niger*	Zinc acetate	80–130	Rod and cluster	Collection and drying at 150°C for 48 h	Antibacterial, antioxidant, and anticancer	Gao et al. (2019)
*Cordyceps militaris*	Zinc nitrate hexahydrate	10.15	Flower	Centrifugation, washing and drying at 60°C	Photocatalytic	Li et al. (2019)
*Fusarium keratoplasticum*	Zinc acetate	10–42	Hexagonal	Collection and drying at 150°C for 48 h	Antibacterial and anticancer	Mohamed et al. (2019)
*A. niger*	Zinc acetate	8–38	Nano-rod	Collection and drying at 150°C for 48 h	Antibacterial and anticancer	
*Alternaria tenuissima*	Zinc sulphate	15.45	Spherical	Ultra-centrifugation, washing and drying at 50°C in hot air	Antimicrobial, antioxidant, anticancer and photocatalytic	Abdelhakim et al. (2020)
*Penicillium corylophilum*	Zinc acetate dihydrate	9–51	Spherical	Centrifugation, washing and oven-drying at 80°C for 48 h	Photocatalytic	Fouda et al. (2020)
*Periconium* sp.	Zinc nitrate	16–78	Quasi spherical	Drying at 125°C for 12 h and calcination at 700°C for 4 h in muffle furnace	Antimicrobial and antioxidant	Ganesan et al. (2020)
*Agaricus bisporus*	Zinc acetate dehydrate	<40 nm	Hexagonal	Filtration, washing and drying at 80°C in hot air oven	Antibacterial and antioxidant	Singh and Dutta (2020)
*Trichoderma harzianum,* and *T. reesei*	Zinc nitrate hexahydrate	60–70	Crystal planes	Calcination at 700°C for 2 h	Antibacterial	Shobha et al. (2020)
*Xylaria acuta*	Zinc nitrate hexahydrate	34–55	Hexagonal	Calcination at 700°C for 2 h	Antimicrobial and anticancer	Sumanth et al. (2020)
*Acremonium potronii*	Zinc acetate hexahydrate	13–15	Spherical	Washing, centrifugation and drying at 150°C for 30 min	Photocatalytic	Ameen et al. (2021)
*A. niger*	Zinc acetate dihydrate	35	Spherical	Centrifugation, washing and drying	Antibacterial and anticancer	Es-haghi et al. (2021)
*A. niger*	Zinc acetate	20	Hexagonal	Washing and centrifugation	Antimicrobial and anticancer	Mekky et al. (2021)
*P. chrysogenum*	Zinc acetate dihydrate	9–35	Hexagonal	Separation and drying at 80°C for 48 h	Antimicrobial	Mohamed et al. (2021)
*A. terreus*	Zinc acetate	30.45	Almost spherical with irregular margins	Ultra-centrifugation, washing and drying at 50°C	Antimicrobial and Antioxidant	Mousa et al. (2021)
*A. niger*	Zinc nitrate	82–176	Irregular, individual, and quaternary	–	Antibacterial and Wound healing	Rasha et al. (2021)
Yeast						
*Pichia fermentans*	Zinc oxide	–	Smooth, and elongated	–	Antimicrobial	Chauhan et al. (2015)
*P. kudriavzevii*	Zinc acetate dihydrate	~10–61	Hexagonal	Centrifugation and drying at 150°C for 6 h	Antibacterial, Antioxidant, and Anticancer	Moghaddam et al. (2017)
Marine yeast	Zinc oxide	86.27	Almost round	–	Antioxidant	Aswathy et al. (2017)

## 3. Proposed mechanisms of microbe-mediated ZnO-NPs synthesis

**Figure 1:**
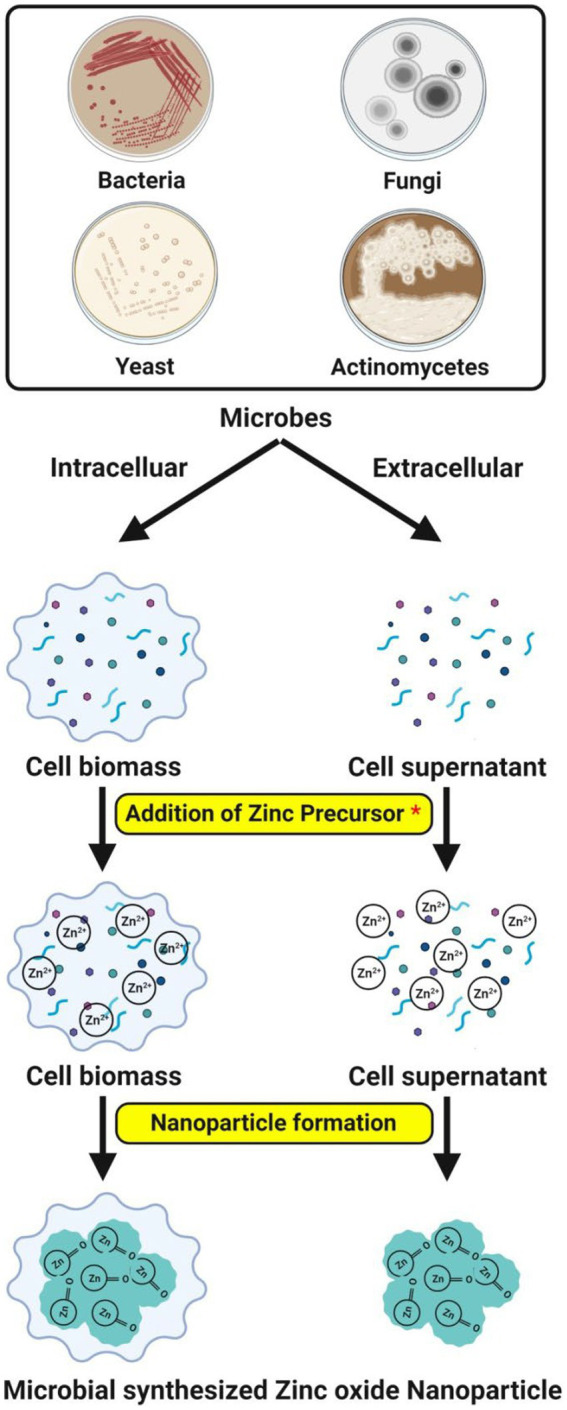
Possible mechanisms of microbe-mediated ZnO-NPs synthesis.

Usually, the microbes have the inherent ability to synthesize the ZnO-NPs directed *via* intra or extracellular pathways. Among the two, synthesis of the nanoparticles through extracellular mode is more favorable and has been extensively employed than intracellular synthesis since it can be utilized to synthesize large extent and involves a considerably simple downstream processing protocol that avoids several synthesis steps as well as easy cell separation and industrialization (Kundu et al., 2014; Busi et al., 2016; Sumanth et al., 2020). To obtain NPs with purity, the recovery of intracellular synthesized NPs requires extra purification steps, such as collecting cell biomass and ultrasonication for cell lysis (Yusof et al., 2020b). It has been well documented that the pH affects the synthesis of NPs through surface or intra or extracellular. Although microbial components, such as proteins, enzymes, and other substances, are essential to the synthesis process, only a few chemical elements are involved in NP production (Figure 1).

By giving Pi (π) electrons from carbonyl groups, bioactive chemicals, such as amino acids and polyphenolic compounds, serve as effective reducing agents during ZnO-NPs synthesis. Following the reduction of zinc ions (Zn^2+^) to zero-valent zinc atoms (Zn^0^), the zinc complex is broken down into ZnO-NPs during annealing. The ZnO nano-rods formed are stabilized by certain extracellular proteins secreted by the bacteria to detoxify the stress condition (Tripathi et al., 2014). The investigations showed that the protein’s amino acids interact with Zn^2+^ ions to create NPs, showing that the protein’s natural state is not necessary to create ZnO-NPs (Jain et al., 2013). The interaction between hydrogen bonds may explain the disruption of non-polar hydrophobic molecules during heating. As a result, zinc ions were exposed to amino acids, leading to the formation of ZnO-NPs. Microbial-secreted amino acids have hydroxyl groups that allow the complexation of zinc ions, hydrolysis, and synthesis of ZnO-NPs via thermal decomposition. The amino acids assist in the stability of ZnO-NPs by aggregation inhibition and control of crystal growth (Moghaddam et al., 2017).

With the aid of microbes and water molecules in an acidic milieu, zinc’s complexation commenced the ZnO-NP production process. The development of the zinc aqua-hydroxo complex, which later changed into ZnO-NPs, was caused by the zinc aqua complex accepting an electron from the deprotonated carboxyl group of bioactive molecules (such as enzymes and peptidoglycan) released by the bacteria (Król et al., 2018). In some cases, the specific organic functional groups present on the surface of the cell wall serve as the building blocks for a non-enzymatic synthesis that aids in reducing the zinc ions. The organic molecules (as reducing agents) are released from the microbial cell due to cell membrane rupture and cell lysis during the heat-killed process. The zinc ions made more organic molecules available, leading to a higher reduction (Yusof et al., 2019). The size, chemical composition, shape, solubility, dispersion factor and surface area of NPs have all significantly impacted their biological responses. The NPs synthesis depends on physicochemical factors such as temperature, pH, precursor concentration, age of microbes, reaction time, stirring, and irradiation, which affect the particle size, monodispersity, shape and yield (Jain et al., 2020).

### 3.1. Mechanism of microbe-mediated intracellular ZnO-NP synthesis

The microbial cell wall structure and ionic charges play a crucial role in the intracellular synthesis pathway of NPs. This technique sends zinc ions into the microbial cell to combine with other molecules, including enzymes and coenzymes, to produce ZnO-NPs. The microbial cell wall comprises various polysaccharides and proteins that activate metal ion binding sites. Heavy metal ions have been demonstrated to pose a significant threat to microbes as they react to the metal stress by penetrating or absorbing the metal ions onto the cell wall structure *via* electrostatic interactions (Selvarajan and Mohanasrinivasan, 2013). It is because specific enzymes, polypeptides, and cysteine on the microbial cell membrane contain negatively charged carboxylate groups that draw metal ions. Likewise, the trapped ions are reduced into elemental atoms by NADH-dependent reductase, wherein an electron carrier enmeshed in the plasma membrane transfers an electron from NADH to the latter (Kundu et al., 2014). Finally, the nuclei transform into NPs that amass in the cytoplasm or periplasmic region bordered by the cell wall. The greatest contributors to NP stability in cells are the proteins, peptides, and amino acids cysteine, tryptophan, and tyrosine (Balraj et al., 2017).

**Figure 2:**
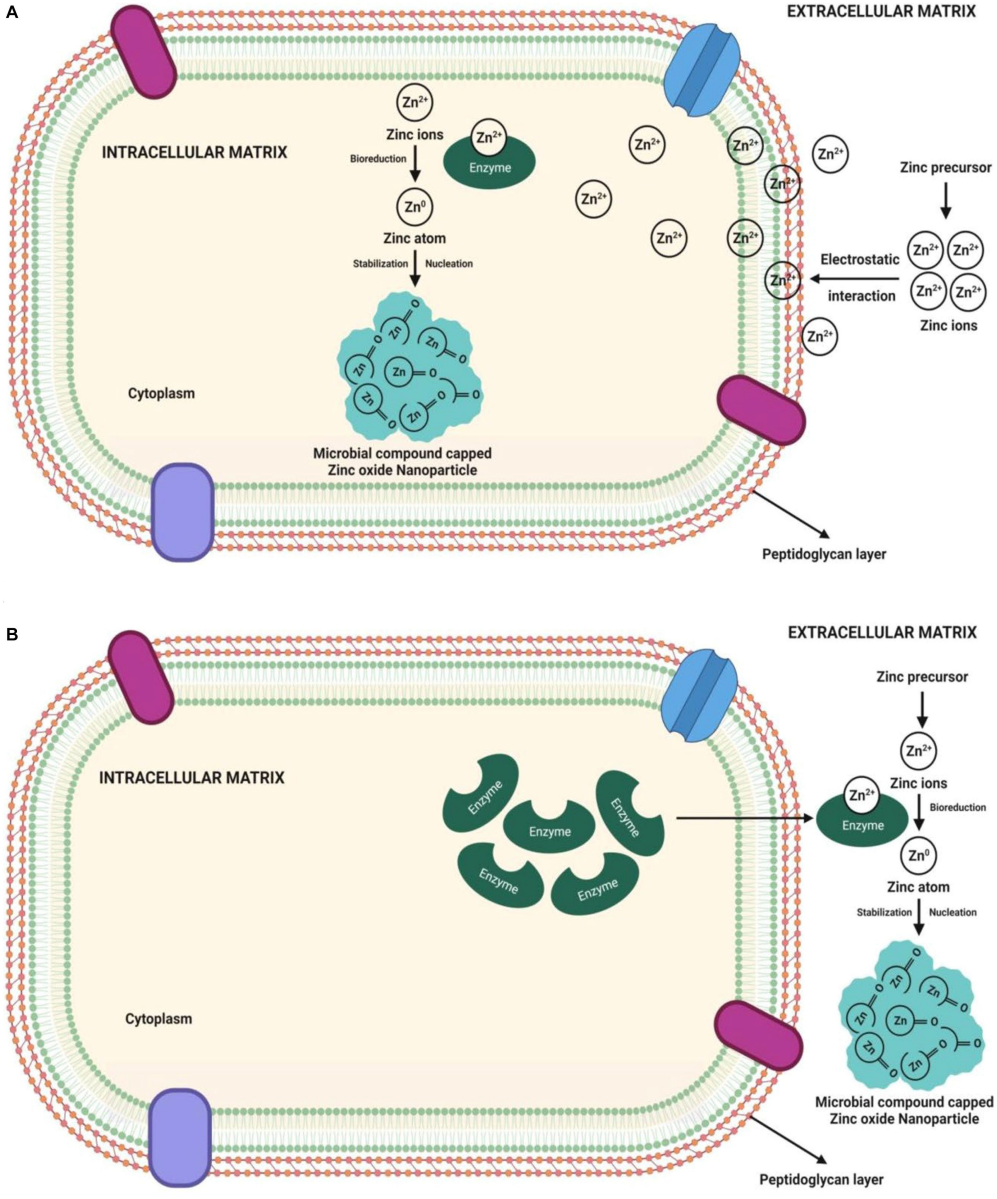
Pictorial representation of mechanisms of microbe-mediated **(A)** intracellular ZnO-NP synthesis and **(B)** extracellular ZnO-NPs synthesis.

The intracellular synthesis mechanism involves the electrostatic transfer of zinc ions into the microbial cell, where the zinc ions (Zn^2+^) are reduced to zinc atoms (Zn^0^) by cell wall enzymes, which subsequently expand the nuclei to form the ZnO-NPswithin the periplasmic space of the cell wall or cytoplasm (Tripathi et al., 2014; Król et al., 2018). The NPs formed are intracellularly penetrated out of the cell. Ultrasonication is required to obtain the pure NPs from intracellular synthesis. The mechanism of microbe-mediated intracellular ZnO-NP synthesis is depicted in Figure 2A. The pH dependent membrane-bound oxidoreductases of probiotic bacteria *Lactobacillus sporogens* employed to synthesize the ZnO-NPs were active at low pH, suggesting a low pH environment required for ZnO NPs synthesis. Similarly, Selvarajan and Mohanasrinivasan (2013) reported that the biosynthesized ZnO-NPs might have resulted from carbon source-dependent rH2 and pH-sensitive membrane-bound oxidoreductases in *L. plantarum* culture solution.

### 3.2. Mechanism of microbe-mediated extracellular ZnO-NP synthesis

Contrary to intracellular synthesis, the extracellular synthesis mechanism involves either enzymatic synthesis on the microbial cell membrane or the release of microbial enzymes (such as cofactor NADH and NADH-dependent enzymes) as extracellular enzymes into the growth medium. The electron transfer from NADH through NADH-dependent reductase (like nitrate reductase enzyme) started the bioreduction of Zn^2+^, which then reduced to Zn^0^, resulting in the ZnO-NP formation (Kundu et al., 2014; Saravanan et al., 2018; Shaaban and El-Mahdy, 2018). Microbes release extracellular proteins that act as capping agents for NPs stability (Balraj et al., 2017). The synthesis of NPs is demonstrated by the appearance of white precipitation in the media. The participation of bacterial extracellular enzymes/ proteins not only allows for the ZnO-NP synthesis but these enzymes/ proteins also control the size and stability of NPs (Kundu et al., 2014; Chauhan et al., 2015; Shaaban and El-Mahdy, 2018). Figure 2B shows a pictorial representation of the microbe-mediated extracellular ZnO-NP synthesis mechanism. The extracellular ZnO-NP synthesis from the culture filtrate of *Aspergillus niger* would be important to identify the responsible reducing agents (Ibrahem et al., 2017; Kalpana et al., 2018). Bacterial secreted metabolites outside the cell carry out the extracellular ZnO-NP synthesis. The siderophore pyoverdine (a secondary metabolite) released extracellularly by *Pseudomonas aeruginosa* comprises amino and hydroxamate groups that reduce Zn^2+^ to form ZnO-NPs (Barsainya and Singh, 2018).

## 4. Bacterial-mediated ZnO-NP synthesis

Several microbes have been used for ZnO-NP synthesis, but bacteria are most preferred due to their genetic manipulation capability and ease of handling over other eukaryotic microbes (Jayabalan et al., 2019; Iqtedar et al., 2020; Ahmed et al., 2021). The reproducible bacteria (like lactic acid producing bacteria) have gained more interest in bacterial-mediated NP synthesis because of their high production of diverse enzymes and non-pathogenic nature. The lactic acid bacteria (Gram-positive) has a thick cell wall layer comprising proteins, polysaccharides, lipoteichoic and teichoic acid, etc., which helps in the bioreduction and biosorption of metal ions (Yusof et al., 2020b; Suba et al., 2021). They can also produce exopolysaccharides that defend cells from metal stress and serve as an additional site for metal ion biosorption (Yusof et al., 2020a). Moreover, lactic acid bacteria are recognized as health-beneficial bacteria for their pessimistic electrokinetic potential (Yusof et al., 2020b), which permits them to attract metal ions for the synthesis of NPs under oxidative as well as reductive conditions (Yusof et al., 2020a,b). Selvarajan and Mohanasrinivasan (2013) have reported that moderately stable ZnO-NPs were produced in which the lactic acid bacteria secreted biomolecules acted as capping agents in intracellular synthesis.

## 5. Actinomycetes-mediated ZnO-NP synthesis

Actinomycetes are regarded as superior among commercially important microbial species because of their saprophytic behavior, allowing them to produce various extracellular enzymes and bioactive components. *Streptomyces* sp. is a member of the actinomycetes, recognized for their soil degrading properties and as the potent source of secondary metabolites, especially antibiotics (Rajivgandhi et al., 2022). Many reports have suggested that actinomycetes can synthesize ZnO-NPs *via* extracellular and intracellular methods (Shanmugasundaram and Balagurunathan, 2017; Kalaba et al., 2021; Rajivgandhi et al., 2022) and needs to be exploited further to utilize them as an alternative to plants. The advantage of using actinomycetes for NP synthesis lies in their ability to produce specific enzymes and metabolites that can control the size, shape, and stability of the NPs formed. It offers potential advantages in tailoring the properties of ZnO-NPs for specific biological applications.

## 6. Fungal-mediated ZnO-NP synthesis

The mechanical method for producing ZnO-NPs from the fungal culture supernatant or biomass is similar to bacterial-mediated synthesis. But, the fungal-mediated ZnO-NP synthesis is a potential strategy owing to their greater metal tolerance, excellent metal binding capacity, metal bioaccumulation ability and higher productivity over bacteria (Baskar et al., 2013; Gao et al., 2019; Sumanth et al., 2020). In addition, the fungi efficiently secrete an excess of extracellular redox enzymes and proteins as bioactive phytochemicals to the culture media than bacteria, which helps reduce the zinc ions (Zn^2+^) into ZnO-NPs in greater quantities (Fouda et al., 2018; Ganesan et al., 2020). The fungal extracellular enzymes and proteins secreted in the media, which act as reducing and capping agents, are bound and encapsulated on the surface of ZnO-NPs, ensuring their stability without agglomeration (Raliya and Tarafdar, 2013; Sarkar et al., 2014).

The fungal strains are typically grown in sterilized media and incubated at the appropriate temperature to prepare the fungal extracts. The fungal metabolite-enriched cell-free supernatant is then harvested by filtration and centrifugation for further ZnO-NP synthesis (Ibrahem et al., 2017; Abdelhakim et al., 2020; Ganesan et al., 2020). The ability of fungal cell-free extracts to potentially cap and reduce the size of ZnO-NPs formed in a definite size and shape was demonstrated. Thus, the shape of fungal-mediated ZnO-NPs synthesized depends on the species type (Mohamed et al., 2019). The plausible mechanism of fungal-mediated ZnO-NPs synthesis has been postulated that the synthesis process involves the initial transformation of zinc acetate (as a precursor) into ZnO-NPs, followed by capping fungal extracellular proteins on the surface of NPs (Kadam et al., 2019).

## 7. Yeast-mediated ZnO-NP synthesis

Like fungi, yeast has been shown to synthesize the ZnO-NPs because of their better zinc stress tolerance. Specific yeast strains are chosen for their ability to interact with zinc ions and facilitate the reduction of zinc ions to form ZnO-NPs. The yeast cells act as a biological reducing agent, converting the zinc ions in the solution into ZnO-NPs through a reduction reaction. The process is usually carried out under controlled conditions to regulate the size and shape of the NPs. The yeast is preferred due to its availability, ease of handling, and biocompatibility. Moghaddam et al. (2017) have reported that ZnO-NPs (~10–61 nm) formed from *Pichia kudriavzevii* yeast strain were found to depend on the reaction time, which plays a main role in the size, distribution and shape of NPs. In addition, studies have revealed that ZnO-NP synthesized from *P. fermentans* extracellularly possessed potent inhibitory effects against various pathogenic bacteria and fungi, thereby contributing to the beneficial effect of the pharmaceutical industry. Due to the toxic metals absorbing and accumulating ability, the diverse yeast species could be used as carriers for synthesizing the ZnO-NPs (Chauhan et al., 2015; Aswathy et al., 2017).

## 8. Applications of microbial-mediated ZnO-NPs

The microbial-mediated ZnO-NPs possess prospective biomedical applications, especially antimicrobial, antioxidant, anticancer, wound healing, drug delivery system, photocatalytic activity, etc., which are described below.

### 8.1. Antibacterial activity

The microbial-mediated ZnO-NPs could be emerged as potential antimicrobial agents mostly because of their unique physiochemical properties in combating a wide range of bacterial pathogens (Moghaddam et al., 2017; Saravanan et al., 2018). Some bacteria can synthesize various antimicrobial chemicals known as bacteriocin (Perez et al., 2014). The microbial-derived bacteriocin might act as a reducing agent in the ZnO-NP synthesis and a capping agent for binding to the NPs surface, thereby increasing their antibacterial properties (Sidhu and Nehra, 2019). The microbial-mediated ZnO-NPs possess more than one mechanism responsible for their antibacterial activity. The possible bactericidal mechanism proposed that the smaller NPs have a higher surface reactivity to penetrate the cells and release free Zn^2+^ from ZnO-NPs easily. The release of Zn^2+^ ions on the biomolecules (DNA/ proteins/ enzymes) found in bacterial cells is one of the major antibacterial mechanisms. It is well known that the formed ZnO-NPs disrupt numerous bacterial cell functions such as metabolism, active transport, and enzyme activity and subsequently induce bacterial cell death (Rauf et al., 2017; Taran et al., 2018; Iqtedar et al., 2020).

**Figure 3:**
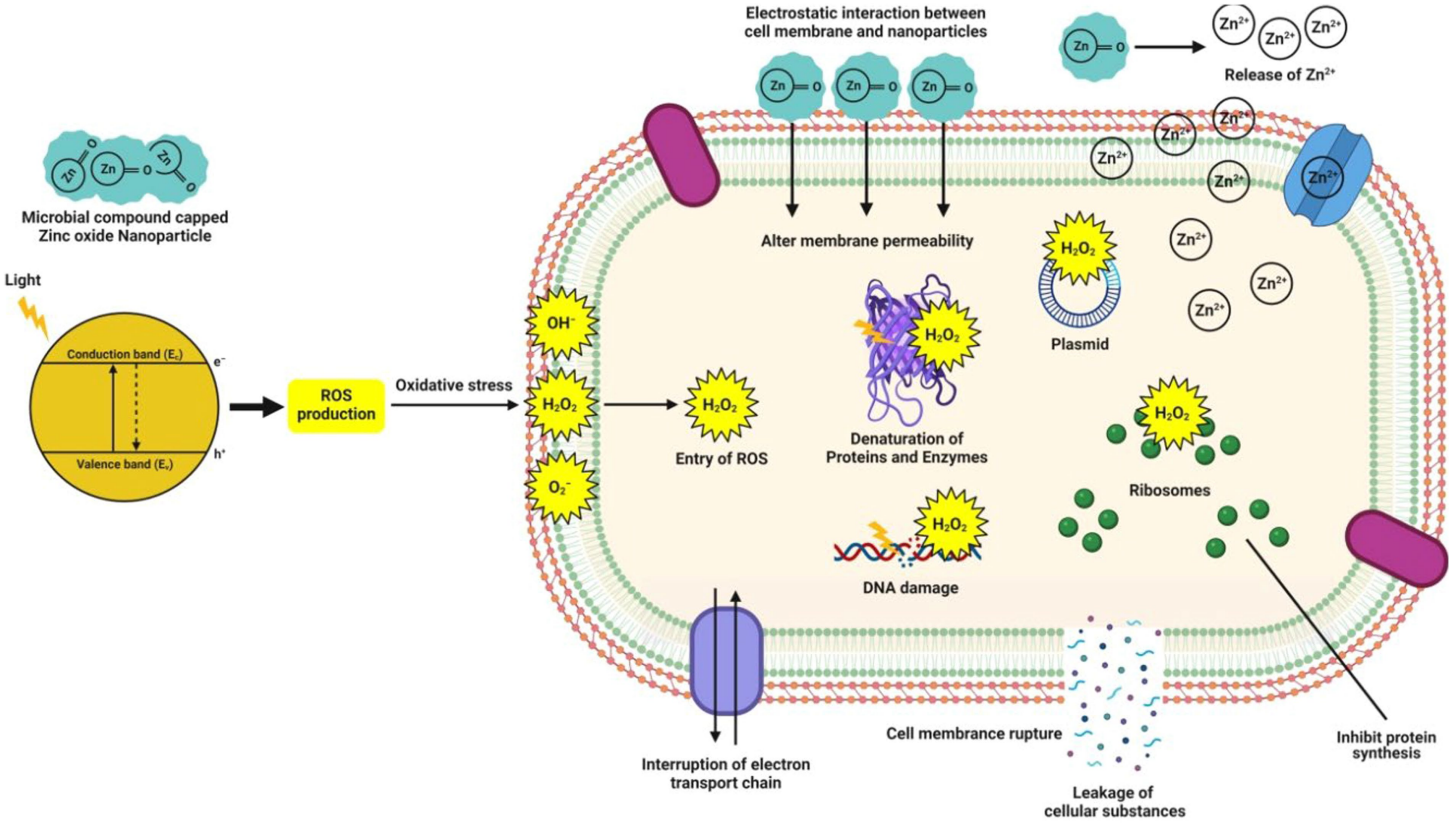
Mechanisms predicted during the expression of antibacterial potential of microbe-mediated ZnO-NPs.

While the ZnO-NPs frequently exploit the production of reactive oxygen species (ROS) [such as superoxide anion (O_2_), hydroxyl ion (OH), and hydrogen peroxide (H_2_O_2_)], when they are exposed to UV light to kill bacteria by inducing oxidative stress and cell death (Rehman et al., 2019). These ROS are generated when hydroxyl groups on the NPs surface react with water (H_2_O) to produce hydroxyl radicals (OH^−^) and hydrogen ions (H^+^), which then produce superoxide anion (O_2_^−^) in the presence of oxygen (O_2_) (Kalpana et al., 2018). Eventually, the bacterial cell membrane barrier is breached by the H_2_O_2_, damaging cellular elements (such as DNA, proteins, and lipids), leading to cell injury and death (Król et al., 2018; Abdelhakim et al., 2020). However, due to their negative charge, OH^−^ and O_2_^−^ are only found on the surface of bacteria and cannot enter the cell membrane except for H_2_O_2_ (Sumanth et al., 2020). The electrostatic adherence of the ZnO-NPs to the bacterial cell membrane is another mechanism underlying the antibacterial potential of these particles. Because ZnO-NPs include amine surface groups, their positive zeta potential facilitates their electrostatic adherence to the negatively charged bacterial cell membrane, which allows NPs to enter the bacterial cells. This direct contact can potentially compromise the integrity of the bacterial cell and rupture the plasma membrane structure, leading to intracellular content leakage and cell death (Jayaseelan et al., 2012; Taran et al., 2018). Figure 3 illustrates the potential antibacterial processes of microbe-mediated ZnO-NPs.

Due to the structure of their cell walls, Gram-negative bacteria are more susceptible to the microbe-mediated ZnO-NPs, while in contrast, Gram-positive bacteria have an extra outer cell membrane that contains lipopolysaccharides that can enhance the outer membrane barrier characteristics, exert a potent aversion to the NPs, and increase their resistance to ZnO-NPs (Gao et al., 2019; Yusof et al., 2020b). The size, composition and shape of NPs influenced their biological activity (i.e., antibacterial activity) because different particle surfaces have varied thicknesses of surface atoms and electronic structures, resulting in various physical and chemical properties (Mohamed et al., 2019). The findings of Mousa et al. (2021) suggested that the ZnO-NPs synthesized using the endophytic *A. terreus* confirmed the broad spectrum of antibacterial activity against four different human pathogenic bacteria (such as *Escherichia coli*, *Klebsiella pneumoniae*, *P. aeruginosa* and *Staphylococcus aureus*) with the recorded MIC value of 100 μg/mL. Suba et al. (2021) have reported that the ZnO-NPs biosynthesized from *Lactobacillus* spp. extract was found to show a strong microbicidal effect with the maximum zone of inhibition ranging from 20 to 24 mm against *Clostridium difficile*, *C. perfringens*, *E. coli*, and *Salmonella typhi*. Recently, Rajivgandhi et al. (2022) suggested that the ZnO-NPs biosynthesized from an actinomycete, *Streptomyces enisocaesilis* possessed antibacterial properties against multi-drug resistant *K. pneumoniae* at increasing concentrations. Additionally, Table 2 lists the antibacterial effectiveness of microbe-mediated ZnO-NPs against bacterial pathogens.

**Table 2:** Microbe-mediated ZnO-NPs exhibiting potential Antibacterial properties.

Microbes	Pathogen	MIC	Reference
Bacteria			
*A. hydrophila*	*Aeromonas hydrophila*; *E. coli*; *S. aureus*; *Enterococcus faecalis; P. aeruginosa* and *Streptococcus pyogenes*	1.2–25 μg mL^−1^	Jayaseelan et al. (2012)
*L. sporogens*	*S. aureus*	20 mM	Mishra et al. (2013)
*S. ureilytica*	*E. coli* and *S. aureus*	–	Dhandapani et al. (2014)
*R. pyridinivorans*	*Staphylococcus epidermidis*	–	Kundu et al. (2014)
*A. schindleri*	*E. coli* and *Salmonella enterica*	100 μg mL^−1^	Busi et al. (2016)
*Streptomyces* sp.	*E. coli* and *Bacillus subtilis*	100 μg mL^−1^	Balraj et al. (2017)
*S. thalpophilum*	*P. aeruginosa* and *Enterobacter aerogens*	0.3 g disc^−1^	Rajabairavi et al. (2017)
*S. aureus*	*S. aureus*; MRSA 1; MRSA 2; *E. faecalis*; *P. aeruginosa*; *S. epidermidis*; *Listeria monocytogenes*; *Klebsiella pneumonia*; *E. coli*; MREC 1 and MREC 2	32–256 μg mL^−1^	Rauf et al. (2017)
*P. aeruginosa*	*S. aureus*; *Bacillus* sp. and *E. coli*	100 μg well^−1^	Barsainya and Singh (2018)
*L. paracasei*	*S. aureus* and *Acinetobacter baumannii*	–	Król et al. (2018)
*B. megaterium*	*Helicobacter pylori* strains	16–17 μg mL^−1^	Saravanan et al. (2018)
*H. elongata*	*E. coli* and *S. aureus*	0.01–0.1 M	Taran et al. (2018)
*P. putida*	*A. baumannii*; *P. otitidis*; *P. oleovorans*; *E. faecalis* and *B. cereus*	30–120 μg μL^−1^	Jayabalan et al. (2019)
*B. haynesii*	*E. coli* and *S. aureus*	4–16 mg mL^−1^	Rehman et al. (2019)
*B. cereus*	*E. coli*; *S. aureus* and *Salmonella typhi*	0.6 μg mL^−1^	Iqtedar et al. (2020)
*S. nematodiphila*	*Xanthomonas oryzae*	100 μg mL^−1^	Jain et al. (2020)
*L. plantarum*	*E. coli*; *Salmonella* sp.; *S. aureus* and *S. epidermidis*	625–2,500 μg mL^−1^	Yusof et al. (2020b)
*B. cereus*	*Burkholderia glumae* and *B. gladioli*	50 μg mL^−1^	Ahmed et al. (2021)
Actinomycetes			
*Streptomyces* sp.	*B. subtilis*; *S. aureus*; *K. pneumoniae*; *E. coli*; *E. aerogenes*; *S. typhi* and *Proteus vulgaris*	50 μg mL^−1^	Shanmugasundaram and Balagurunathan (2017)
*S. plicatus*	*Erwinia amylovora*	15.6 μg mL^−1^	Kalaba et al. (2021)
*S. enisocaesilis*	*K. pneumoniae*	50–250 μg mL^−1^	Rajivgandhi et al. (2022)
Fungi			
*A. fumigatus*	*K. pneumoniae*; *P. aeruginosa*; *E. coli*; *S. aureus* and *B. subtilis*	250–1,000 μg mL^−1^	Rajan et al. (2016)
*A. niger*	*S. aureus* and *E. coli*	1–1.5 mg mL^−1^	Ibrahem et al. (2017)
*A. terreus*	*P. aeruginosa*; *S. aureus*; *B. subtilis* and *E. coli*	250–500 μg mL^−1^	Fouda et al. (2018)
*A. niger*	*E. aerogenes*; *E. coli*; *K. pneumoniae* and *P. aeruginosa*	500 μg mL^−1^	Gao et al. (2019)
*F. keratoplasticum*	*E. coli*; *S. aureus*; *B. subtilis* and *P. aeruginosa*	250–500 ppm	Mohamed et al. (2019)
*A. niger*			
*A. tenuissima*	*P. aeruginosa, K. pneumoniae, E. coli* and *S. aureus*	100–200 μg mL^−1^	Abdelhakim et al. (2020)
*Periconium* sp.	*S. aureus* and *E. coli*	50 μg/mL	Ganesan et al. (2020)
*A. bisporus*	*B. subtilis* and *E. coli*	10–100 μg mL^−1^	Singh and Dutta (2020)
*T. harzianum* and *T. reesei*	*Xanthomonas oryzae*	25–50 μg mL^−1^	Shobha et al. (2020)
*X. acuta*	*E. coli*; *B. cereus*; *S. aureus* and *P. aeruginosa*	15.6–31.3 μg mL^−1^	Sumanth et al. (2020)
*A. niger*	*E. coli*	–	Es-haghi et al. (2021)
*A. niger*	*A. baumannii*; *K. pneumoniae*; *P. aeruginosa*; *E. coli*; *S. aureus* and *S. haemolyticus*	12.5–50 μg mL^−1^	Mekky et al. (2021)
*P. chrysogenum*	*B. subtilis*; *S. aureus*; *S. typhimurium*; *E. coli* and *P. aeruginosa*	2–3 mg mL^−1^	Mohamed et al. (2021)
*A. terreus*	*S. aureus*; *P. aeruginosa*; *E. coli* and *K. pneumoniae*	100 μg mL^−1^	Mousa et al. (2021)
*A. niger*	*K. pneumoniae*	0.7 mg mL^−1^	Rasha et al. (2021)
Yeast			
*P. fermentans*	*P. aeruginosa*	–	Chauhan et al. (2015)
*P. kudriavzevii*	*B. subtilis*; *S. aureus*; *S. epidermidis*; *S. marcescens* and *E. coli*	40–100 μg mL^−1^	Moghaddam et al. (2017)

### 8.2. Antifungal activity

**Figure 4:**
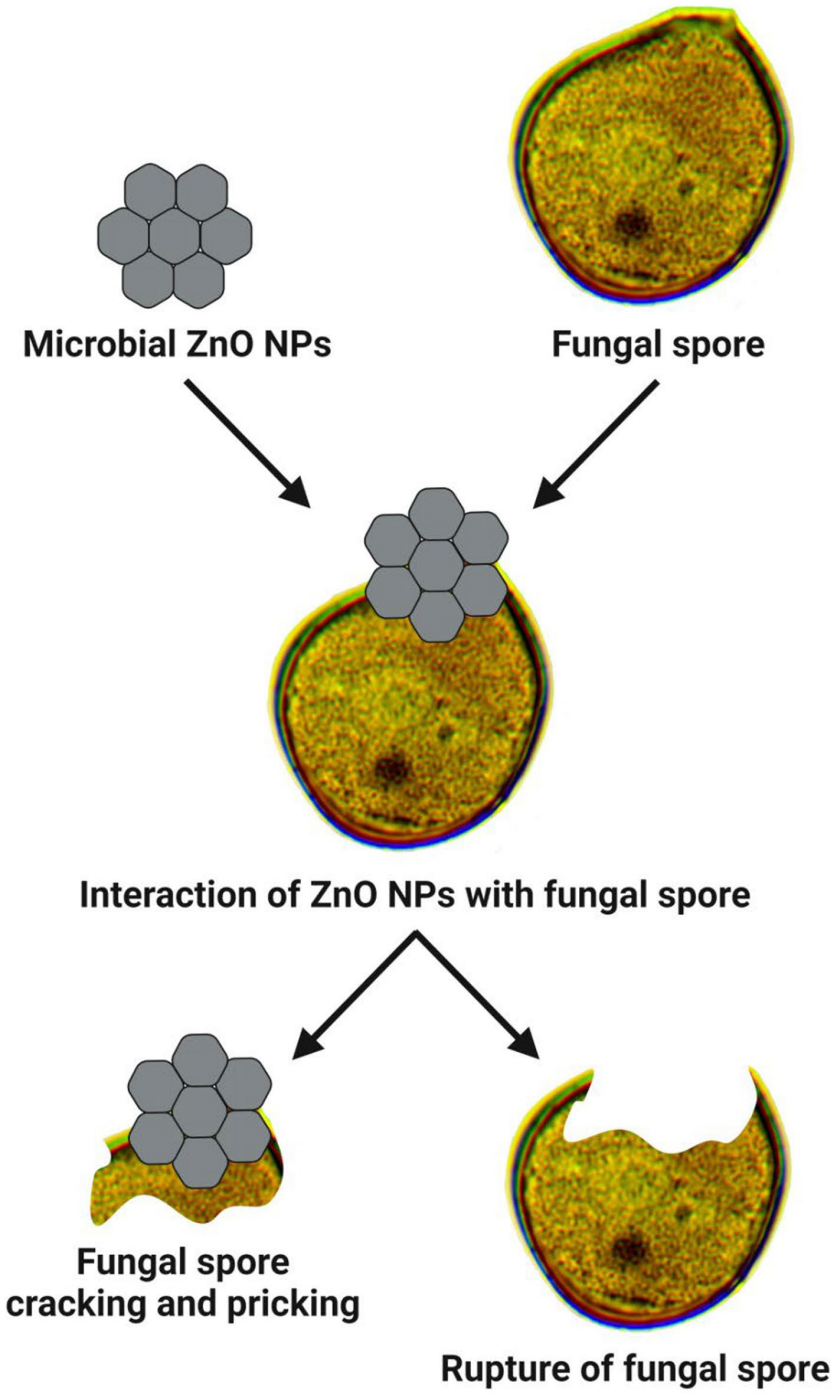
Mode of action involved in antifungal properties of microbe-mediated ZnO-NPs.

The mechanism of antifungal activity of microbe-mediated ZnO-NPs was similar to the antibacterial mechanism. ROS production could promote the antifungal activity of ZnO-NPs as the hydroxyl radicals are produced in the water suspension of NPs, which trigger the breakdown of fungal cell membranes and inhibit cell growth (Jain et al., 2020). The release of Zn^2+^ ions by ZnO-NPs, when they come into close contact with the fungal cell membrane could be another mechanism of action. The positive charge of Zn^2+^ ions is easily attracted to the negative charge of the cell membrane, which reacts further with the sulfhydryl groups of protein inside the cell membrane and damages the synthetase activity. As a result, the fungi lose their cell division and growth, which finally leads to cause their cell death (Figure 4; Jayaseelan et al., 2012; Chauhan et al., 2015; Jain et al., 2020).

Jain et al. (2020) have demonstrated that the synthesized ZnO-NPs using *Serratia nematodiphila* significantly offered antifungal activity against the phytopathogenic *Alternaria* sp. in a dose-dependent manner. Sumanth et al. (2020) have revealed that the extracellular ZnO-NPs synthesized from *Xylaria acuta* extract exhibited antifungal activity against *A. flavus*, *Cladosporium cladosporioides*, *Fusarium oxysporum*, and *Phomopsis* sp., with the higher inhibition percentage of fungal mycelia in a dose-dependent manner. Mohamed et al. (2021) reported that the ZnO-NPs synthesized using *Penicillium chrysogenum* showed antifungal potential against phytopathogenic fungi (such as *A. terreus*, *F. oxysporum*, *F. solani*, and *Sclerotium sclerotia*) at 10 mg/mL. Recently, Mousa et al. (2021) revealed that the synthesized ZnO-NPs from the endophytic *A. terreus* showed promising antifungal potential against two human pathogenic fungi (such as *A. brasiliensis* and *Candida albicans*) and two plant pathogenic fungi (such as *Alternaria alternata* and *F. oxysporum*) with the recorded MIC value of 100 μg/mL. Besides, Suba et al. (2021) have reported that the ZnO NPs biosynthesized from *Lactobacillus* spp. extract also exhibited the antifungal effect with the maximum inhibition zone (i.e., 18–21 mm) against *A. flavus* and *C. albicans*. The antifungal activity of microbe-mediated ZnO-NPs against various fungal pathogens is listed in Table 3.

**Table 3:** Microbe-mediated ZnO-NPs exhibiting potential antifungal properties.

Microbes	Pathogen	MIC	Reference
Bacteria			
*A. hydrophila*	*A. flavus*; *A.niger* and *C. albicans*	0.9–25 μg mL^−1^	Jayaseelan et al. (2012)
*P. aeruginosa*	*Rhizoctonia solani*; *Fusarium* sp., and *Penicillium* sp.	100 μg well^−1^	Barsainya and Singh (2018)
*S. nematodiphila*	*Alternaria* sp.	250 μg mL^−1^	Jain et al. (2020)
*Lactobacillus* spp.	*C. albicans* and *A. flavus*	–	Suba et al. (2021)
Actinomycetes			
*S. plicatus*	*A. flavus*; *A. niger, F. oxysporum*; *F. moniliform* and *A. alternata*	62.5–500 μg mL^−1^	Kalaba et al. (2021)
Fungi			
*A. tenuissima*	*C. albicans*; *A. solani*; *A. niger* and *F. oxysporum*	50–200 μg mL^−1^	Abdelhakim et al. (2020)
*Periconium* sp.	*C. albicans*	40 μg mL^−1^	Ganesan et al. (2020)
*X. acuta*	*A. flavus*; *Phomopsis* sp.*, F. oxysporum* and *Cladosporium cladosporioides*	100–400 μg mL^−1^	Sumanth et al. (2020)
*A. niger*	*A. niger*; *P. marneffei*; *C. glabrata* and *C. parapsilosis*	12.5–25 μg mL^−1^	Mekky et al. (2021)
*P. chrysogenum*	*F. solani*; *F. oxysporum*; *Sclerotium sclerotia* and *A. terreus*	10 mg mL^−1^	Mohamed et al. (2021)
*A. terreus*	*C. albicans*; *A. brasiliensis*; *A. alternata* and *F. oxysporum*	100 μg mL^−1^	Mousa et al. (2021)
Yeast			
*P. fermentans*	*Fusarium* sp.; *Ganoderma* sp. and *A. terreus*	–	Chauhan et al. (2015)

### 8.3. Antioxidant activity

**Figure 5:**
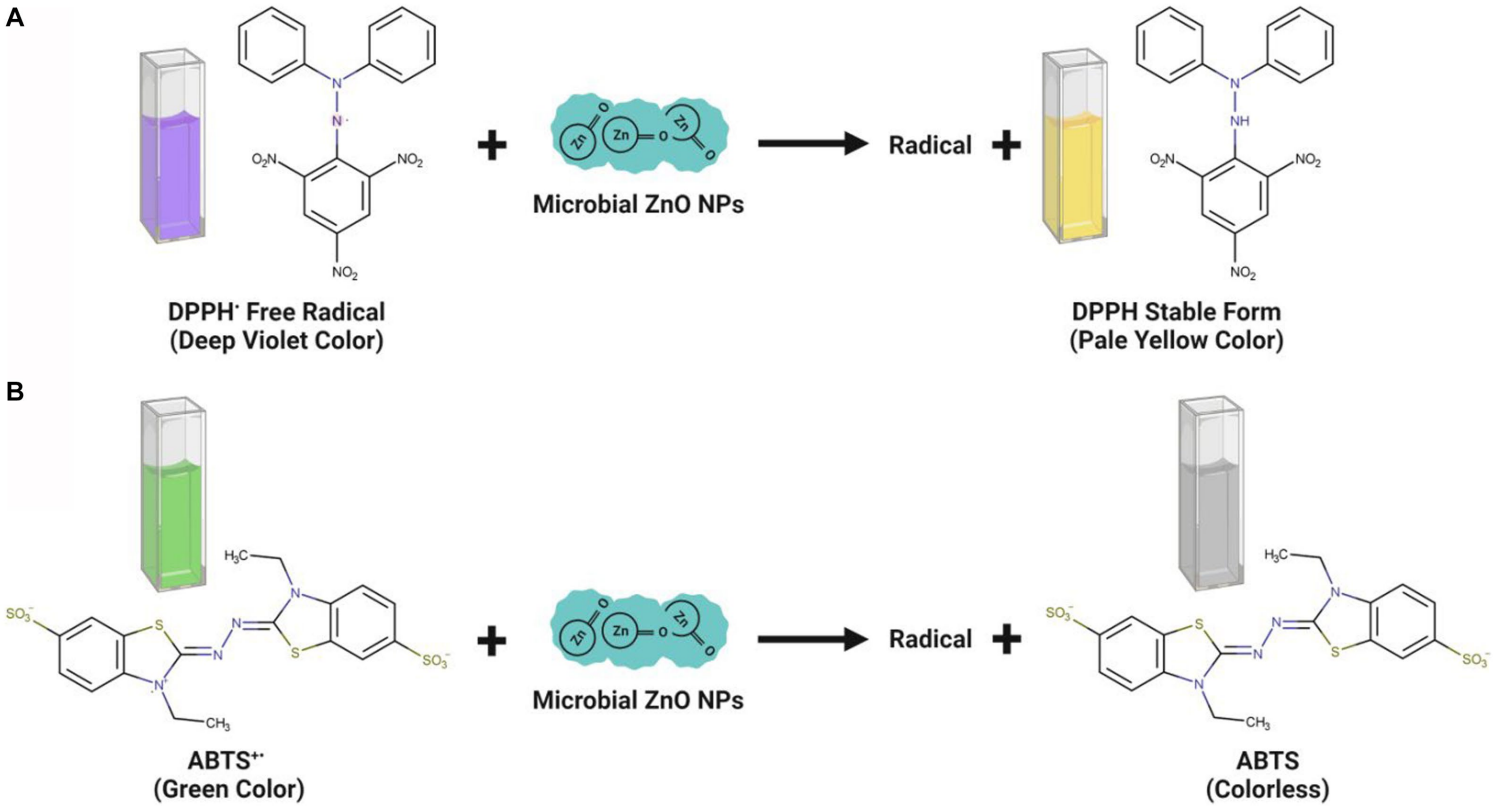
Antioxidant mechanisms observed upon interaction of microbe-mediated ZnO-NPs during DPPH **(A)** and ABTS **(B)** assay.

The microbe-mediated ZnO-NPs showed promising antioxidant properties, paving the way for their use as a new antioxidant source. The antioxidant potential is mainly due to the inhibition and neutralization of DPPH and ABTS free radical formation (Moghaddam et al., 2017; Abdelhakim et al., 2020). The DPPH is a stable nitrogen-centered, lipophilic free radical extensively employed to assess antioxidant activity. It can easily accept an electron or hydrogen radical from the corresponding donor to become a stable diamagnetic molecule. The odd electron located at the nitrogen atom in DPPH is reduced to hydrazine by receiving a hydrogen atom from the antioxidants. The DPPH solution color gradually changes from its characteristic deep purple/ violet color to pale yellow in the presence of ZnO-NPs. The DPPH radical scavenging activity was calculated by measuring the DPPH solution color change from deep purple to pale yellow spectrophotometrically at 517 nm, showing a strong absorption band (Figure 5A; Gao et al., 2019).

The findings of the DPPH experiment revealed that the ZnO-NPs samples effectively inhibited free radicals. The many phytochemicals attached to the NP surface may play a significant role in the antioxidant activity of microbe-mediated ZnO-NPs samples (Ganesan et al., 2020). The DPPH radical scavenging activity also depends on the solubility of ZnO-NPs. The scavenging activity of ^•^OH radicals was also evaluated to determine whether the synthesized ZnO-NPs could protect deoxyribose sugar from damage by scavenging ^•^OH radicals (Singh et al., 2014). The functional groups and bioactive components in the fungal biomass may be responsible for the significant antioxidant property and free radical quenching capacity of microbe-mediated ZnO-NPs (Gao et al., 2019). The bioactive components are thought to be involved in hydrogen atom donation to prevent the free radical process.

Conversely, the ABTS assay has also proved that the microbe-mediated ZnO-NPs have a strong potential for scavenging activity of the harmful free radicals (Figure 5B). Abdelhakim et al. (2020) have suggested that ZnO-NPs synthesized using the culture filtrate of endophytic fungus (*A. tenuissima*) exhibited promising antioxidant activity with 50% inhibitory concentration (IC_50_) of 102.13 μg/mL, which was mainly due to the inhibition and neutralization of DPPH free radical formation. Ganesan et al. (2020) have reported that the endophytic fungal (*Periconium* sp.) extract mediated ZnO-NPs exhibited good antioxidant properties at 100 μg mL^−1^ concentration with 85.52% of DPPH free radical quenching. Mousa et al. (2021) have indicated that the ZnO-NPs synthesized from the endophytic *A. terreus* had a promising antioxidant activity with the recorded DPPH radical scavenging activity (IC_50_ value) of 108.67 μg mL^−1^. Besides, the antioxidant activity of microbe-mediated ZnO-NPs is represented in Table 4.

**Table 4:** Microbe-mediated ZnO-NPs exhibiting potential antioxidant properties.

Microbes	Half-maximal inhibitory concentration (IC_50_)	Maximum activity	Reference
Bacteria			
*P. aeruginosa*	200 μg mL^−1^	40–70%	Singh et al. (2014)
Actinomycetes			
*Streptomyces* sp.	500 μg mL^−1^	75–80%	Shanmugasundaram and Balagurunathan (2017)
Fungi			
*A. niger*	1,000 μg mL^−1^	50%	Es-haghi et al. (2019)
*A. niger*	100 μg mL^−1^	57.74–73.58%	Gao et al. (2019)
*A. tenuissima*	102.13 μg mL^−1^	50%	Abdelhakim et al. (2020)
*Periconium* sp.	100 μg mL^−1^	85.52%	Ganesan et al. (2020)
*A. bisporus*	100 μg mL^−1^	67–79%	Singh and Dutta (2020)
*A. terreus*	108.67 μg mL^−1^	50%	Mousa et al. (2021)
Yeast			
*P. kudriavzevii*	5.26–25.46 μg mL^−1^	50%	Moghaddam et al. (2017)
Marine yeast	80–100 μg mL^−1^	48.95–54.1%	Aswathy et al. (2017)

### 8.4. Anticancer activity

**Figure 6:**
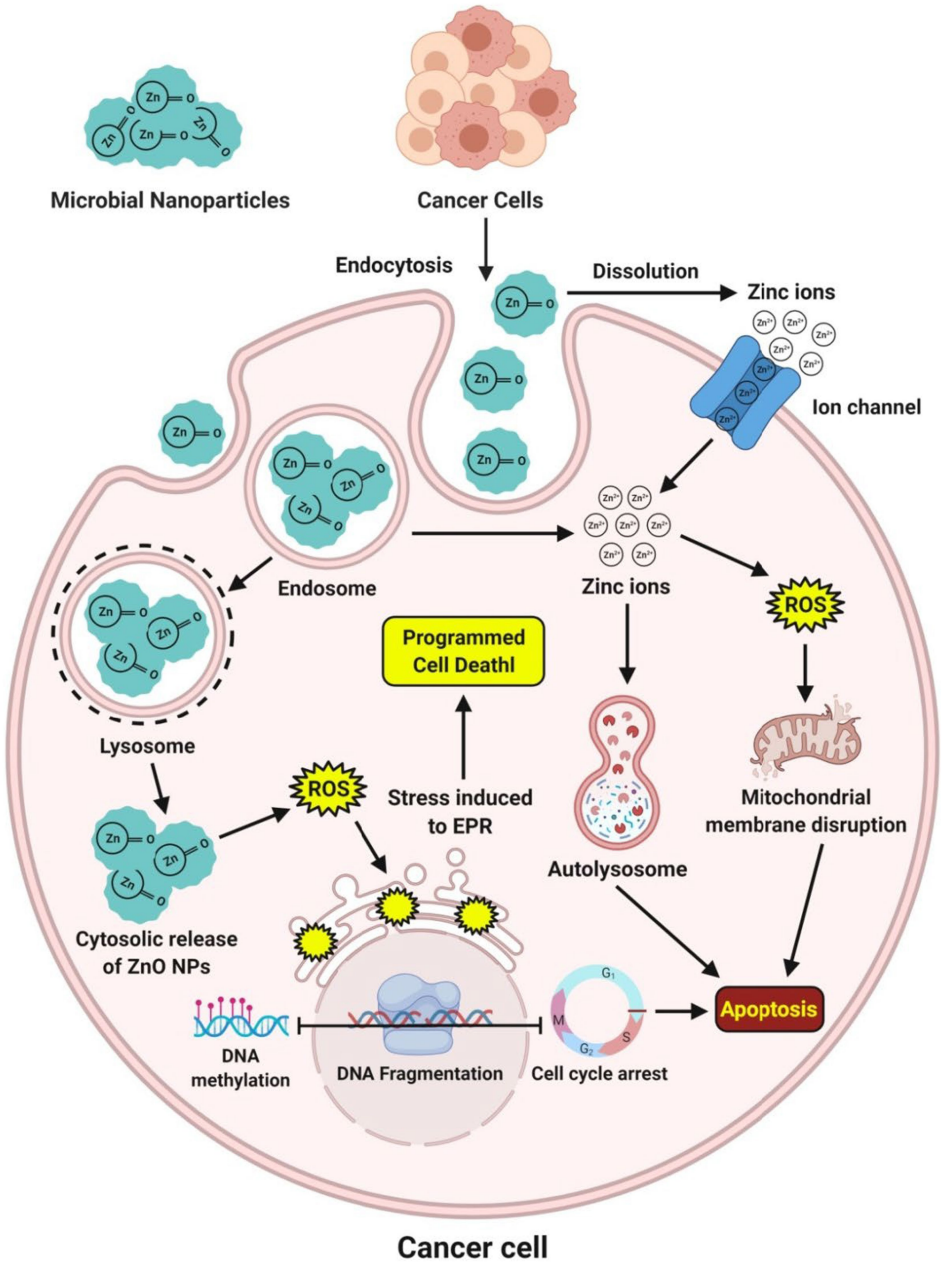
Mechanism of action observed during the expression of anticancer potential of microbe-mediated ZnO-NPs.

Microbe-mediated ZnO-NPs are gaining much more interest in exploring as an excellent anticancer therapeutic candidate with low toxicity than chemically synthesized ZnO-NPs (Gao et al., 2019). The ZnO-NPs positive surface charge interacts with the cancerous cells’ negative charge cell membranes, resulting in the membrane’s rupture, oxidative stress, and cell death. The interaction between the positive surface charge of ZnO-NPs and the negatively charged cancer cell membrane caused the cell membrane to rupture, allowing ROS to enter the cancer cell and causing oxidative stress and cell death. An extensive study has reported that microbe-mediated ZnO-NPs can be selectively targeted and display remarkable apoptotic features in the cytoplasm and nucleus. They regulated oxidative stress, cell cycle progression, DNA replication, DNA repair, and finally induced apoptosis in a dose-dependent manner in many cancer cell lines (Figure 6). Cell dynamic behavior is expected to be based on cell sorting and varies in response to different shapes of NPs. However, interestingly the cytotoxicity of microbe-mediated ZnO-NPs is shape-dependent and dose-dependent (Mohamed et al., 2019).

Abdelhakim et al. (2020) have reported that the ZnO-NPs synthesized from the culture filtrate of endophytic fungus, *A. tenuissima*, were found to be more active against both malignant MCF-7 and HepG-2 cancer cell lines as well as the non-malignant Hbf-4 cell line with the IC_50_ concentrations from 16.87–55.76 μg mL^−1^. Sumanth et al. (2020) have suggested that the extracellular ZnO-NPs synthesized from *X. acuta* extract showed anticancer activity at 1 μg mL^−1^ concentration, which internalized and distributed without disturbing the morphology of cancer cells. Moreover, Suba et al. (2021) have reported that the MTT test using biosynthesized ZnO-NPs from *Lactobacillus* spp. extract confirmed the excellent biocompatibility activity and exhibited a potential toxic effect of 54.16 μg mL^−1^ in the human colon cancer (HT-29) cell line. Additionally, the anticancer activity of microbe-mediated ZnO-NPs is summarized in Table 5.

**Table 5:** Anticancer activity of microbe-mediated ZnO-NPs.

Microbes	Toxicity against cell lines	Activity (IC_50_ Value)	Reference
Bacteria			
*R. pyridinivorans*	Colon Cancer (HT-29)	–	Kundu et al. (2014)
*Streptomyces* sp.	Lung Cancer (A549)	15.6 μg mL^−1^	Balraj et al. (2017)
*Lactobacillus* spp.	Colon Cancer (HT-29)	54.16 μg mL^−1^	Suba et al. (2021)
Actinomycetes			
*Streptomyces* sp.	Human Osteosarcoma (MG63), Vero cells	200 μg mL^−1^	Shanmugasundaram and Balagurunathan (2017)
Fungi			
*A. alternata*	Human Lymphocyte Cells	≥500 μg mL^−1^	Sarkar et al. (2014)
*A. terreus*	Human Colorectal Adenocarcinoma (Caco-2), Normal Vero (kidney of African green monkey), Normal Rat Liver Epithelial (Colne-9)	26.85–79.57 μgmL^−1^	Fouda et al. (2018)
*A. niger*	Human Breast Carcinoma (MCF-7)	38–50 μg mL^−1^	Es-haghi et al. (2019)
*A. niger*	Liver Cancer (HepG2), Human Embryonic Kidney (HEK-293) (non-cancerous)	19.16–26.75 μgmL^−1^	Gao et al. (2019)
*F. keratoplasticum*	Human Colorectal Adenocarcinoma (Caco-2), Normal Vero (kidney of African green monkey), Normal Rat Liver Epithelial (Colne-9)	20.1–104.3 ppm	Mohamed et al. (2019)
*A. niger*		57.6–131 ppm	
*A. tenuissima*	Normal Human Melanocytes (HFB-4), Hepatocelluar Carcinoma (HepG-2), Human Breast Carcinoma (MCF-7)	16.87–55.76 μg mL^−1^	Abdelhakim et al. (2020)
*X. acuta*	Human Mammary Gland Carcinoma (MDA-MB 134)	1 μgmL^−1^	Sumanth et al. (2020)
*A. niger*	Human Breast Carcinoma (MCF-7), Skin Fibroblast Cells	–	Es-haghi et al. (2021)
*A. niger*	Wi-38 Cells	~800.42 μg mL^−1^	Mekky et al. (2021)
Yeast			
*P. kudriavzevii*	Vero Cells	190–238 μg mL^−1^	Moghaddam et al. (2017)

The biocompatibility of microbe-mediated ZnO-NPs is essential, especially when exploring their potential anticancer activity. Biocompatibility refers to the ability of the NPs to interact with biological systems without causing harm or adverse effects. In cancer therapy, it is crucial to ensure that the NPs selectively target cancer cells while sparing normal cells to minimize side effects; thus, researchers must assess their effects on both cancerous and normal cells (Sumanth et al., 2021). The ideal scenario is to find a therapeutic concentration of ZnO-NPs that effectively kills cancer cells while having minimal toxicity toward healthy cells. Achieving this selective targeting can be challenging, as many anticancer agents can also affect normal cells. It is important to note that while microbe-mediated ZnO-NPs may offer promising anticancer properties, thorough biocompatibility studies are necessary before considering their use at clinical levels (Rauf et al., 2017; Murali et al., 2021b; Faisal et al., 2022).

### 8.5. Drug delivery system

**Figure 7:**
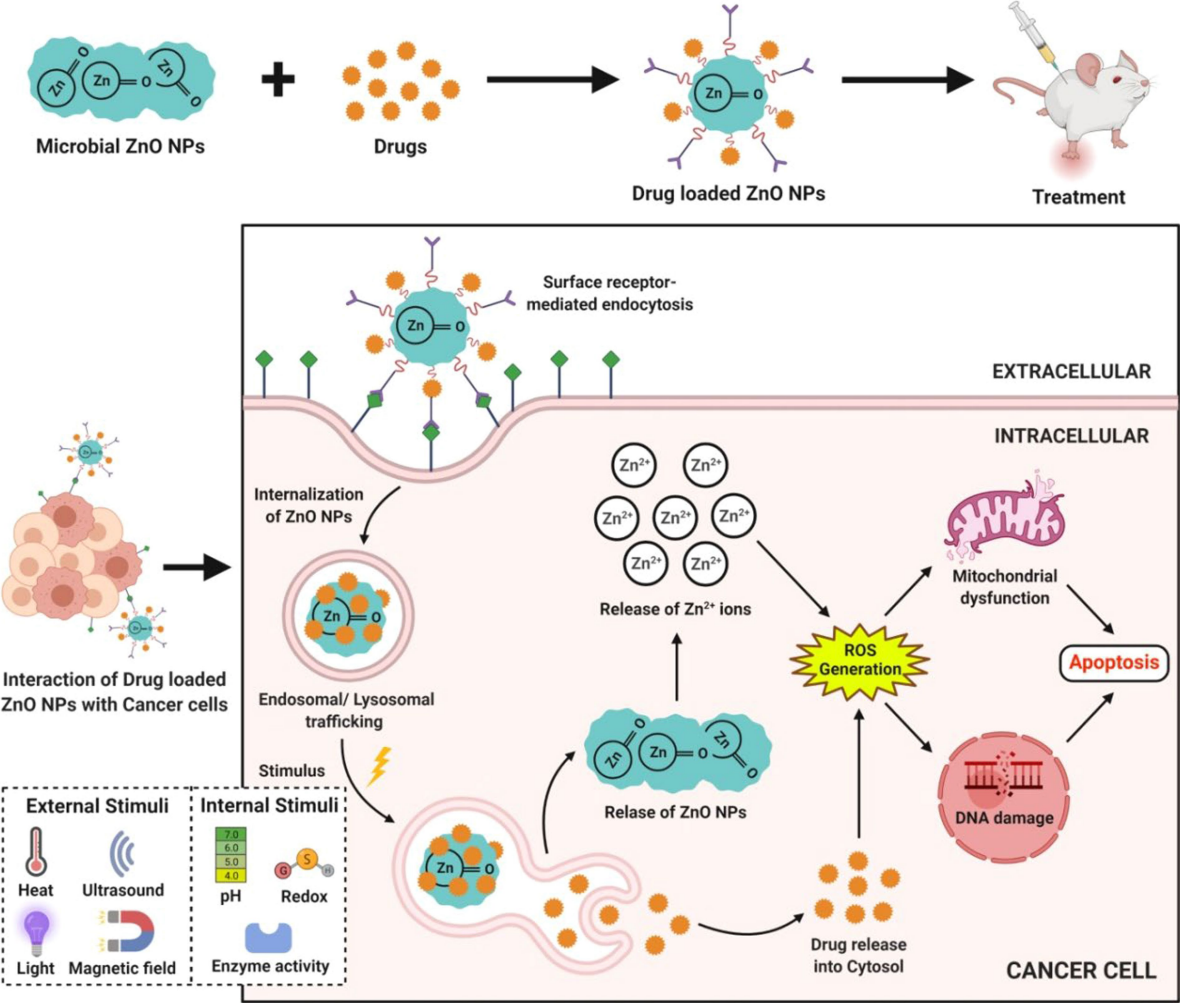
Action of microbe-mediated ZnO-NPs during drug delivery.

In addition to their anticancer properties, the microbe-mediated ZnO-NPs are being used as promising drug delivery vehicles because of their biocompatibility and extremely low toxicity to normal cells. The mechanism behind the cytotoxicity of microbe-mediated ZnO-NPs against cancer cells is the targeted drug delivery to the cancer cells with decreased toxicity to non-target organs. The microbe-mediated ZnO-NPs are utilized to deliver targeted drugs with the anticancer agent released once it reaches the diseased zone of the body. ZnO-NPs are also employed in targeted drug delivery to extend the drug retention time. The anthraquinone-loaded ZnO-NPs synthesized from *Rhodococcus pyridinivorans* exhibited a preferential ability to kill HT-29 cancer cells compared with the normal peripheral blood mononuclear cells and anthraquinone loaded ZnO-NPs could be used as anticancer drug delivery vehicles (Figure 7; Kundu et al., 2014). These findings highlight the exciting potential of microbe-mediated ZnO-NPs as a viable tool for delivering bioactive anticancer drugs over time. Thus, they can be used as safe biological and exact drug delivery vehicles for cancer treatment in the near future.

### 8.6. Wound healing

Additionally, the biologically synthesized ZnO-NPs using *A. niger* improve the carbapenem-resistant *K. pneumonia* infected wound healing property in experimental rats (Rasha et al., 2021). This recent advance shows that this microbial nanotherapeutics is promising prospects for treating burn wounds.

### 8.7. Photocatalytic activity

**Figure 8:**
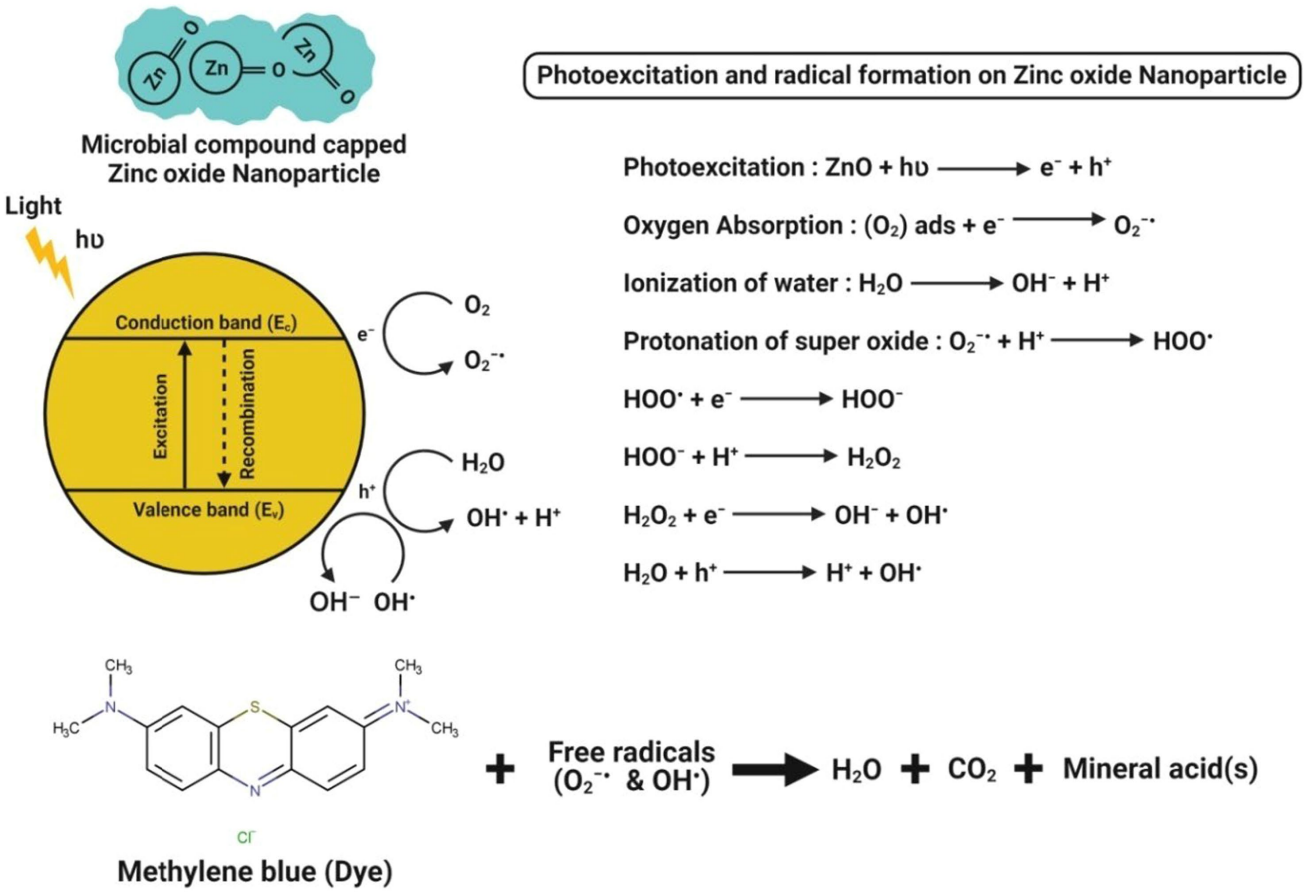
Chemical interactions observed during the photocatalytic activity of microbe-mediated ZnO-NPs.

The microbe-mediated ZnO-NPs could also be applied as an excellent photocatalyst in degrading many organic dyes under UV irradiation (Tripathi et al., 2014). The proposed mechanism of photocatalytic activity of microbe-mediated ZnO-NPs is illustrated in Figure 8. When light with energy greater than or equal to the bandgap illuminates ZnO-NPs, an electron (e^−^) can be excited from the valence band (VB) to the conduction band (CB). At the same time, a hole (h^+^) is simultaneously generated in the VB, thereby forming electron–hole (e^−^h^+^) pairs. The hole oxidizes the water molecules adsorbed on the surface of NPs into hydroxyl radicals (^•^OH) and hydrogen ions (H^+^), while the photoexcited electron reacts with dissolved oxygen (O_2_) to form superoxide radicals (O_2_^•–^). The reaction between H^+^ and O_2_^•–^ further produces hydroperoxyl radicals (^•^OOH), which can produce hydrogen peroxide (H_2_O_2_). These radicals have extremely high oxidative properties that easily degrade the toxic organic compounds into carbon dioxide and water molecules (Fouda et al., 2020).

Li et al. (2019) have reported that *Cordyceps militaris* fungus-mediated ZnO-NPs had the degradation ability of hazardous dye (like methylene blue) about 97% within 180 min irradiation of UV light. Abdelhakim et al. (2020) have suggested that ZnO-NPs synthesized from the culture filtrate of endophytic *A. tenuissima* further efficiently showed their photocatalytic behavior by the complete (100%) degradation of methylene blue dye in a concentration-dependent manner after 20 min exposure to sunlight. Jain et al. (2020) demonstrated that the ZnO-NPs synthesized from *S. nematodiphila* showed ~90% of methyl orange degradation after 80 min of UV light irradiation, which was evident by the visual color observation and absorbance peak intensity at 463 nm. Recently, Siddique et al. (2021) efficiently bioprospecting *Pseudochrobactrum* sp. for the green synthesis of ZnO-NPs and reported that the biosynthesized ZnO-NPs significantly showed a better photocatalytic degradation potential of various dyes (*viz.,* methylene blue, 4-nitriophenol, brilliant blue R, brilliant yellow, reactive black 5, and reactive red 120) after 10 h exposure to sunlight as compared to the chemically synthesized ZnO-NPs. This higher photocatalytic degradation might be due to the relatively smaller size, more stability and higher surface area of the biosynthesized NPs. Moreover, the photocatalytic activity of microbe-mediated ZnO-NPs is represented in Table 6.

**Table 6:** Photocatalytic activity of microbe-mediated ZnO-NPs.

Microbes	Dye degraded	Irradiation type and Intensity range	Irradiation time	pH range	Degradation efficiency (%)	Reference
Bacteria						
*R. pyridinivorans*	Malachite green	UV irradiation at 300–800 nm	2 h	–	95%	Kundu et al. (2014)
*B. licheniformis*	Methylene blue	UV irradiation at 365 nm	60 min	–	83%	Tripathi et al. (2014)
*S. nematodiphila*	Methyl orange	UV light	80 min	8.06	~90%	Jain et al. (2020)
Fungi						
*A. niger*	Bismarck brown	UV light at 365 nm	–	–	90%	Kalpana et al. (2018)
*C. militaris*	Methylene blue	UV light	180 min	–	97%	Li et al. (2019)
*A. tenuissima*	Methylene blue	Sunlight	20 min	–	100%	Abdelhakim et al. (2020)
*P. corylophilum*	Methylene blue	Visible light	85 min	–	98%	Fouda et al. (2020)
*A. potronii*	Methylene blue	UV–vis light	30 min	Nearly 6.5	93%	Ameen et al. (2021)
*Pseudochrobactrum* sp.	Methylene blue, 4-nitriophenol, brilliant blue R, brilliant yellow, reactive black 5, reactive red 120	Sunlight	280 min – 10 h	–	44.9–95.2%	Siddique et al. (2021)

## 9. Toxicological effects

The toxicological effects of microbe-mediated ZnO-NPs are an important area of research and concern. When the NPs are synthesized using microbes such as bacteria, actinomycetes, fungi, or yeast, their potential toxicity can differ from conventionally produced NPs due to size, shape, surface charge, and chemical composition variations. It is important to note that the toxicological effects of NPs can be highly dependent on factors such as specific microbial synthesis methods, distinctive characteristics of NPs, dose, exposure route, and the vulnerability of target organisms. Research on the toxicity of microbial-mediated ZnO-NPs is ongoing, and it is crucial to conduct comprehensive studies to understand and assess their potential risks fully. It was reported that the larger NPs also tend to accumulate in the kidneys for a longer period due to the slower glomerular filtration excretion mechanisms, and this prolonged retention might result in organ damage (Rauf et al., 2017; Sumanth et al., 2021; Faisal et al., 2022). Furthermore, the different morphologies (such as nanorods, nanoflowers, nanosheets and nanoplates) of NPs also contribute to their toxicity due to their larger surface area. The fungal-mediated ZnO-NPs were significantly found to show *in vitro* cytotoxicity and genotoxicity in human lymphocyte cells, wherein they demonstrated a concentration-dependent decrease in mitochondrial activity at ≥0.5 mg mL^−1^ and induced DNA fragmentation at 1 mg mL^−1^ (Sarkar et al., 2014). Many NPs toxicity studies have focused on the plants due to their critical functions in ecosystems as primary producers of organic compounds from atmospheric or aqueous carbon dioxide. Güllüce et al. (2020) have reported that the ZnO-NPs synthesized by *Rhodococcus erythropolis* using the precursors (such as zinc sulfate heptahydrate, zinc nitrate hexahydrate, zinc chloride and zinc acetate) were found to show their toxicological potentials on *Triticum aestivum*, where they significantly affected the seed germination and seedling growth along with their genotoxic potentials. If released into the environment, the microbe-mediated ZnO-NPs may interact with living organisms and ecosystems, potentially causing ecological toxicity and disrupting natural processes. Therefore, it is further necessary to investigate any potential long-term toxicological effects of microbe-mediated ZnO-NPs on plants, animals and human health due to these multiple uses in numerous industries. To ensure more effective and safe use of ZnO-NPs, it is also essential to adopt responsible manufacturing practices, perform rigorous toxicity assessments, and follow safety assessments and regulatory guidelines. In addition, Table 7 provides the advantages and disadvantages of microbe-mediated synthesis of ZnO-NPs.

**Table 7:** Advantages and disadvantages of microbial-mediated ZnO-NPs over plant-mediated synthesis.

**Advantages**	**Disadvantages**
Clean, non-toxic, environmentally acceptable and easily scaled up for large-scale synthesis. Hydrophilic in nature, highly stable and disperse uniformly in water. Show the biocompatibility properties and have the potential to be used in versatile biomedical applications. Various enzymes, biomolecules and proteins in microbes are used as the capping agents for forming multiple-sized NPs. Demonstrated the capability to act as a natural nano-factory. Microbial synthesis of ZnO-NPs offers an advantage over plants as they can be easily reproduced. Microbes exhibit high metal resistance due to the adsorption of metals and their chelation by intracellular and extracellular proteins.	Isolation and screening of potential microbes and vector construction is time-consuming. Requires highly aseptic conditions and special maintenance. Microbes might lose their ability to synthesize NPs as a consequence of mutations. Isolation of microbes and their culture media enhances the cost-competitive feasibility. The recovery process of ZnO-NPs during intracellular synthesis requires additional steps for microbial cell disruption to get the intracellular ZnO-NPs. The synthesis process involves the use of chemicals for growth media.

## 10. Future prospects

The major drawback of microbe-mediated synthesis of ZnO-NPs is that the microbes may lose their capacity to synthesize NPs because of mutations over time. In addition, not all microbes can produce ZnO-NPs; thus, those potential microbes need to be explored through rigorous screening programs. It is still unknown how exactly microbes synthesize NPs through extracellular and intracellular processes. There is a lack of knowledge regarding the reducing and capping agents involved in bioreduction and stabilization, respectively, during NPs synthesis. Uncertainty exists regarding the identity of reducing and capping agents and their function in influencing both the size and shape of NPs. Particularly because of the requirement of a complete aseptic environment and specific maintenance, the microbe-mediated NPs are not suitable for large-scale production. In the future, extensive optimisation studies are required before scaling up the industrial production of NPs to understand the impact of every aspect completely. Further, cooperation between major stakeholders in basic sciences, chemical engineering, and industry is required to exploit the microbe-mediated ZnO-NPs economically. Future research studies must address the concentration and duration of exposure required for causing the potential toxicologic effects of ZnO-NPs to understand the therapeutic effects better and prevent unintended cytotoxic effects.

## 11. Conclusion

The present review highlights the need for green synthesis approaches in producing ZnO-NPs due to the environmental pollution caused by conventional chemical synthesis methods. Using biological sources like plants and microbes makes the process more environmentally friendly and safer. The advantages of microbial synthesis include using bioactive metabolites released by microbes as reducing and stabilizing agents, which enhance the biological properties of the ZnO-NPs. The extracellular biosynthesis method is simpler since the enzymes and proteins outside the cells directly reduce or chelate Zn^2+^ ions. In contrast, the intracellular method requires an additional cell lysis step to release the NPs inside the microbe, making it more expensive and time-consuming. Among the microbial synthesis methods, fungal-mediated synthesis shows promise due to its ability to produce more bioactive compounds, while bacteria have an advantage in cell growth activity.

The review also suggests that microbial synthesis has excellent biotechnological potential and could pave the way for large-scale industrial production of ZnO-NPs. Since the biological components are natural, biodegradable, and safer, this approach is expected to be a promising alternative for plant-mediated ZnO-NPs for future industrial and commercial production. However, further research is still needed to understand the biochemical and molecular mechanisms of NP synthesis among different microbes. Investigating these mechanisms could unlock even more potential applications of microbe-mediated ZnO-NPs in various fields, including medicine, industry, environment, and agriculture. Overall, this review emphasizes the importance of green synthesis methods and highlights the promising role of microbe-mediated synthesis in producing ZnO-NPs with exciting applications in diverse areas. It encourages further research in this area to exploit the full potential of these eco-friendly nanomaterials and extend the next level of their applications.

## Author contributions

All authors listed have made a substantial, direct, and intellectual contribution to the work and approved it for publication.

## Conflict of interest

The authors declare that the research was conducted in the absence of any commercial or financial relationships that could be construed as a potential conflict of interest.

## Publisher’s note

All claims expressed in this article are solely those of the authors and do not necessarily represent those of their affiliated organizations, or those of the publisher, the editors and the reviewers. Any product that may be evaluated in this article, or claim that may be made by its manufacturer, is not guaranteed or endorsed by the publisher.
